# Ponatinib for CML patients in routine clinical practice: the PONDEROSA study

**DOI:** 10.1007/s00277-026-06788-6

**Published:** 2026-01-15

**Authors:** Thomas Schenk, Christian Fabisch, Thomas Ernst, Philipp Ernst, Susanne Saussele, Daniela Žáčková, Jiří Mayer, Hana Klamová, Andreas Hochhaus

**Affiliations:** 1 Abteilung Hämatologie und Internistische Onkologie, Klinik für Innere Medizin II, Universitätsklinikum Jena, Jena, Germany; 2 Comprehensive Cancer Center Central Germany Jena/Leipzig – Campus Jena, Jena, Germany; 3 Medizinische Klinik III, Medizinische Fakultät Mannheim der Universität Heidelberg, Mannheim, Germany; 4 Department of Internal Medicine, Hematology and Oncology, University Hospital Brno and Masaryk University, Brno, Czech Republic; 5 Institute of Hematology and Blood Transfusion, Praha, Czech Republic

**Keywords:** Chronic myeloid leukemia, Ponatinib, Registry, Real-world evidence, Dose optimization, Arterial occlusive events

## Abstract

Ponatinib, a third-generation tyrosine kinase inhibitor, is effective in patients with chronic myeloid leukemia (CML), particularly in cases of resistance or *BCR::ABL1* T315I mutation. However, arterial occlusive events (AOEs) remain an important safety concern. The PONDEROSA registry evaluated ponatinib use under routine clinical conditions in Germany and the Czech Republic. This observational cohort study included 99 adult CML patients treated with ponatinib; patient recruitment took place between 2015 and 2022 at 31 centers. The median follow-up was 22 months (range: 1–83). Among the 99 patients (median age 54 years at CML diagnosis), 91.9% were in chronic phase, 4.0% in accelerated phase, and 4.0% in blast phase. The T315I BCR::ABL1 mutation was detected in 19.2%. Ponatinib starting doses were 45 mg/day (32.3%), 30 mg/day (37.3%), or 15 mg/day (29.3%). Adverse events (AEs) were recorded in 64.6% of patients. Severe cardiovascular or cerebrovascular events occurred in 12.1% of patients, with no fatal events observed. Ponatinib was discontinued in 31.3% of patients, mainly due to intolerance or lack of response. 58.6% of patients achieved or maintained at least a major molecular response (MMR), compared to 19.0% at baseline. Disease progression was observed in 14.1% of patients, and 8.1% underwent allogeneic stem cell transplantation. The estimated 2-year progression-free survival and overall survival rates were 84.4% and 85.7%, respectively. The PONDEROSA study confirms the clinical effectiveness of ponatinib in routine practice. Individualized dosing strategies are essential to balance efficacy and cardiovascular safety. Ponatinib remains a valuable bridging therapy for patients requiring allogeneic transplantation.

**Supplementary Information:**

The online version contains supplementary material available at 10.1007/s00277-026-06788-6.

## Introduction

The advent of tyrosine kinase inhibitors (TKIs) has fundamentally transformed the treatment of chronic myeloid leukemia (CML), shifting it from a fatal malignancy to a chronic, manageable disease. For most CML patients, survival rates are now comparable to those of the general population [1]. Despite the success of first- and second-generation TKIs, such as imatinib, dasatinib, and nilotinib, approximately 20–30% of patients require a switch to an alternative TKI due to resistance or intolerance, underscoring the need for additional treatment options [2]. Ponatinib, a third-generation TKI, was developed to address these challenges by targeting resistant *BCR::ABL1* mutations, including the highly resistant T315I mutation [3], which confers resistance to all other currently available TKIs, with the exception of asciminib. Clinical trials such as PACE and OPTIC demonstrated the efficacy of ponatinib in heavily pretreated CML patients, achieving deep and durable responses even in advanced disease settings. In the PACE trial, 60% of evaluable chronic-phase-CML patients achieved a major cytogenetic response (MCyR), with an 82% probability of maintaining the response for 5 years. A complete cytogenetic response (CCyR) was observed in 54% of patients, while 40% achieved a major molecular response (MMR). However, arterial occlusive events (AOEs) emerged as a key safety concern, with a cumulative incidence of up to 25% at five years, including 20% classified as serious [4]. These observations prompted a detailed safety analysis that revealed a dose-dependent relationship: each 15 mg/day reduction in average ponatinib dose was estimated to lower the risk of arterial thrombosis by approximately 33% [5]. To address these concerns, the OPTIC trial was designed to assess different ponatinib starting doses (45 mg, 30 mg, and 15 mg daily), with mandated dose reduction to 15 mg once a *BCR::ABL1* international scale (IS) transcript level of ≤ 1% was achieved. This response-guided strategy demonstrated that initiating treatment at 45 mg and reducing to 15 mg preserved efficacy while reducing the incidence of AOEs compared to continuous high-dose therapy [6, 7], which was confirmed by long-term follow-up over five years [8]. In parallel, asciminib, an inhibitor that selectively targets the ABL myristoyl pocket (STAMP), has emerged as a promising new therapy with a unique mechanism of action. It provides an alternative for patients with cardiovascular risk factors and intolerance to other TKIs [9]. For patients with the T315I mutation, asciminib requires substantially higher doses than for other patient groups. Clinical data from the phase I CABL001X2101 trial showed that most patients with a T315I mutation who achieved a MMR had received doses ≥ 150 mg twice daily​ [10]. A 2-year follow-up analysis from this study, involving 48 patients with T315I-mutated chronic-phase chronic myeloid leukemia (CML-CP), confirmed the efficacy and safety of asciminib at 200 mg twice daily, demonstrating sustained molecular responses [11]. Based on these findings, in the US a recommended asciminib dose of 200 mg twice daily was approved for this patient population [12]. Nevertheless, ponatinib remains an important therapeutic option in the management of T315I-mutated CML [13]. At the time the PONDEROSA registry was initiated, asciminib had not yet been approved, and ponatinib represented the only effective treatment option for many patients with resistant CML. Registry studies conducted in Argentina, Belgium, France, Israel and Italy have confirmed ponatinib’s effectiveness in diverse CML patient populations. These studies demonstrated its ability to achieve molecular responses even in CML patients with advanced disease and extensive prior TKI therapy [14–20]. To build on these findings, the PONDEROSA study evaluates routine clinical outcomes of ponatinib treatment in Germany and the Czech Republic. The study provides region-specific insights into the clinical application of ponatinib and its role in CML management.

## Methods

### Study design

The PONDEROSA registry is a multicenter, observational cohort study conducted in Germany and the Czech Republic. This study combines prospective and retrospective data collection to provide a comprehensive overview of ponatinib use in routine clinical practice. Treatment decisions, including initiation and dose adjustments, were made at the discretion of the treating physician. The study did not require additional diagnostic tests or follow-up visits beyond those performed in routine care. It was conducted in accordance with the Declaration of Helsinki and Good Clinical Practice (GCP) guidelines, with approval from relevant ethics committees. All participants provided written informed consent prior to inclusion.

## Patient population

The registry included adult patients (≥ 18 years) with CML in any phase who initiated ponatinib monotherapy after its approval in Germany as part of routine care. Both prospective and retrospective cohorts were analyzed. Patients were required to provide written informed consent and have a minimum life expectancy of three months. Exclusion criteria included prior treatment with investigational ponatinib, ongoing participation in a clinical trial with an investigational agent, pregnancy or breastfeeding, and inability to provide informed consent.

## Data collection

Data were collected during routine clinical visits, approximately every three months, or as required by standard care. All data were documented using an electronic Case Report Form (eCRF). Retrospective data were extracted from patient medical records. Collected information included demographic details, disease characteristics, previous therapies, ponatinib dosing history, and molecular or cytogenetic responses. Safety data were systematically recorded, with a particular focus on vascular occlusive events. These included arterial and venous thrombotic or occlusive events, such as myocardial infarction, angina pectoris, coronary artery disease, cerebrovascular ischemic events, peripheral artery occlusive disease, retinal vascular thrombosis, and venous thromboembolism. Standardized definitions for arterial occlusive events were applied across all study centers according to the PONDEROSA observational protocol.

## Objectives

The primary objective of this study was to provide an overview of ponatinib use in everyday clinical practice. Specifically, the study aimed to assess:

- Response to treatment, including MMR and deeper responses (MR4, MR4.5).
- Ponatinib dosing patterns, including starting doses, dose adjustments, and treatment duration.
- The incidence of adverse events (AEs), with a focus on cardiovascular and cerebrovascular events.

As secondary objectives, the study also evaluated progression-free survival (PFS) and overall survival (OS).

### Statistical analysis

Descriptive statistics were used to summarize baseline characteristics and treatment patterns.

Given the limited cohort size (*n* = 99), the mixed prospective–retrospective design, and incomplete availability of key baseline variables, no multivariable models were performed because they would have been underpowered and at risk of bias; heterogeneity in data completeness and follow-up between subcohorts further precluded meaningful multivariate testing. Continuous variables were reported as mean, median, and range, while categorical variables were presented as frequencies and percentages. Survival analysis and Kaplan–Meier plots were generated using lifelines software for python (Version v0.30.0) [21].

## Results

### Patients’ characteristics

This study included 99 patients with CML recruited from 27 centers in Germany (*n* = 76 patients) and 4 in the Czech Republic (*n* = 23 patients) between July 2015 and May 2022. The study sites comprised 14 ambulatory care centers, 5 hospitals, and 12 university hospitals. The patient cohort consisted of 44 individuals enrolled in the prospective and 55 in the retrospective arm. The median age at CML diagnosis was 54 years (range, 17–85), and at ponatinib initiation, it was 59 years (range, 21–88). The cohort comprised 42.4% female patients. The median follow-up duration was 22 months (range: 1–83). At treatment initiation, 91 patients (91.9%) were in the chronic phase (CP), 4 (4.0%) in the accelerated phase (AP), and 4 (4.0%) in the blast phase (BP) according to the 2016 WHO classification, including two cases of myeloid and two cases of lymphoid BP. Molecular testing identified a *BCR::ABL1* T315I mutation in 19 patients (19.2%). Among them, two patients harbored additional mutations: one with Y253H and one with E255K (both in combination with T315I). In addition, three patients had other non-T315I mutations, including one with Y253H, one with F317L, and one with a dual mutation of V299L and F359I. Regarding prior therapy, 36 patients (36.3%) had received two prior TKIs, while 44 (44.4%) had received three or more TKIs. The median duration of first-line therapy was 11 months (range: 0.1–119), while the median duration of second-line therapy was 13 months (range: 0.1–206). A total of 43 patients (43.4%) had received imatinib as first-line therapy, whereas 47 patients (47.5%) had been treated with a second-generation TKI (dasatinib or nilotinib) as their initial therapy. Additionally, five patients received interferon as first-line treatment, while in four cases, no information on first-line therapy was available. Ponatinib was commenced after a median duration of 3 years (range: 0–27) following CML diagnosis. Several cardiovascular risk factors were present in the study population. Baseline assessment of modifiable cardiovascular risk factors showed that 42 patients (42.4%) had arterial hypertension, 13 patients (13.1%) had diabetes mellitus, and nine patients (9.1%) had hyperlipidemia. Hypercholesterinemia was reported in five patients (5.1%). The median body mass index (BMI) was 25.3 kg/m² (range: 18.1–39.8 kg/m²), with obesity (BMI ≥ 30 kg/m²) documented in 15 patients (15.1%). This included 10 patients (10.1%) classified as Grade I obesity and five patients (5.0%) as Grade II obesity. Smoking status was reported for 50 patients, of whom 11 patients were former smokers and five patients were current smokers. For 49 patients (49.5%), smoking status was unknown (Table 1). In terms of cardio- and cerebrovascular disease history, coronary artery disease, congestive heart failure, and cardiac arrhythmia were each reported in 4% of patients. Peripheral arterial disease and transient ischemic attacks were present in 3% of cases. Two patients (2%) had a history of myocardial infarction or thrombophilia, while one patient (1%) had experienced embolic events.

**Table 1:** Baseline characteristics and cardiovascular risk factors of the study population (*N* = 99)

Parameter	Value
Total patients	99
Participating sites (Germany / Czech Republic)	31 (27 / 4)
Female	42 (42.4%)
Median age at diagnosis of CML (years, range)	54 (17–85)
Median age at start of ponatinib (years, range)	59 (21–88)
Median follow-up (months, range)	22 (1–83)
**CML Phase (according to WHO 2016 classification)**	
Chronic Phase	91 (91.9%)
Accelerated Phase	4 (4.0%)
Blast Phase	4 (4.0%)
**Mutation Status**	
T315I *BCR::ABL1* mutation	19 (19.2%)
Other *BCR::ABL1* mutations	5 (5.0%)
**Previous TKIs**	
2 Prior TKIs	36 (36.3%)
≥ 3 Prior TKIs	44 (44.4%)
Imatinib as first-line therapy	43 (43.4%)
2nd generation TKI as first-line therapy	47 (47.5%)
**Modifiable cardiovascular risk factors**	
Ex-Smoker	11 (11.1%)
Current smoker	5 (5.1%)
Unknown smoking status	49 (49.5%)
Arterial hypertension	42 (42.4%)
Diabetes mellitus	13 (13.1%)
Body mass index (kg/m², median, range)	25.3 (18.1–39.8)
Obesity grade 1	10 (10.1%)
Obesity grade 2	5 (5.0%)
Hyperlipidemia	9 (9.1%)
Hypercholesterolemia	5 (5.1%)

## Response to prior TKI therapy

Before initiating ponatinib, the best response achieved with prior TKI therapy was at least a MMR in 34 of 78 evaluable patients (43.6%), while 44/78 patients (56.4%) had never reached MMR. In 21 cases, molecular response data were not available. At the last assessment before switching to ponatinib, 15 of 79 evaluable patients (19.0%) maintained at least MMR, whereas 64/79 patients (81.0%) had lost response or never achieved MMR. In 20 cases, molecular response data remained unknown. A deep molecular response (MR4 or better) was observed in 18 of 78 evaluable patients (23.1%) as their best response to prior TKI therapy, while 7/79 patients (8.8%) still had MR4 or better at the last assessment before ponatinib initiation.

### Molecular response under ponatinib treatment

Among patients who continued ponatinib treatment and had available molecular response data, an improvement in response rates was observed over time. At month 12, molecular response data were available for 51 patients, and at month 24, for 38 patients. At month 12, 51.0% of these patients had reached MMR. By month 24, 73.7% of patients with available data had reached MMR, and 44.7% had achieved MR4 or better. Overall, at the last follow-up, 58 of 99 patients (58.6%) had achieved at least an MMR. Response rates were calculated from molecular results documented in the registry; potential overestimation cannot be excluded, as molecular assessments were routinely performed but not always completely reported to the registry. However, given the decreasing number of evaluable molecular response data over time, response rates should be interpreted with caution.

### Ponatinib treatment and safety

The initial ponatinib dose varied among patients, with 29.3% receiving 15 mg/day, 37.3% receiving 30 mg/day, and 32.3% starting at 45 mg/day. A total of 53.5% of patients (*n* = 53) underwent dose modifications, including 43 dose reductions and 18 dose increases. The most common reasons for dose reductions were non-hematologic AEs (41.9%), good treatment response (37.2%), and cytopenia (9.3%). In contrast, dose escalations were primarily due to suboptimal or lack of response (61.1%), followed by good tolerability and the goal of optimizing response (22.2%). After dose reductions, 28 patients continued at 15 mg, 12 patients at 30 mg, one patient at 22.5 mg, and two patients at 7.5 mg/day. Ponatinib discontinuation was reported in 31.3% of patients (*n* = 31), most commonly due to lack of response (54.8%) or non-hematologic adverse events (29.0%). The latter included drug intolerance in four patients, as well as peripheral arterial occlusive disease (*n* = 2), myocardial infarction (*n* = 1), cardiomyopathy (*n* = 1), and cerebral ischemia (*n* = 1). Additional reasons were cytopenia (12.9%) and, in one case, patient preference (3.2%). The median duration of ponatinib therapy before discontinuation was 6 months (range, 0.8–78 months). Two patients (2.0%) were able to stop ponatinib after achieving a deep molecular response and successfully maintained treatment-free remission (TFR) (Table 2). During ponatinib treatment, AEs were recorded in 64 out of 99 patients (64.6%). The most frequently reported AEs included pain (14.5%), general disorders (12.5%), and laboratory abnormalities (11.4%). Cardiovascular and cerebrovascular events were among the notable toxicities. Arterial hypertension and hypertensive crises occurred in 17.2% of patients and accounted for 8.0% of all AEs. Other cardiac AEs were reported in 15 patients (15.2%). Severe (grade 3–4) cardiovascular or cerebrovascular AEs were recorded in 12 patients (12.1%), comprising 14 of 289 AEs (4.8%). These included five events of severe hypertension, three of peripheral artery disease, three strokes, one case of angina pectoris, one NSTEMI, and one severe cardiac arrhythmia. Importantly, no fatal cardiovascular or cerebrovascular AEs were reported.

**Table 2:** Overview of Ponatinib dose modifications and treatment discontinuations (*N* = 99)

Category	*N* (%)
**Initial ponatinib dose**	
15 mg/day	29 (29.3)
30 mg/day	37 (37.3)
45 mg/day	32 (32.3)
Unknown	1 (1)
**Patients with dose modifications**	53 (53.5)
**Reasons for dose reduction** (*N* = 43 documented dose change events)	
Non-hematologic AEs	18 (41.9)
Favorable treatment response	16 (37.2)
Cytopenia	4 (9.3)
Other	5 (11.6)
**Reasons for dose increase** (*N* = 18 documented dose change events)	
Suboptimal or lack of response	11 (61.1)
Good tolerability/response optimization	4 (22.2)
Other	3 (16.7)
**Patients with ponatinib discontinuation**	31 (31.3) *
Reasons for ponatinib discontinuation (*N* = 31)	
Lack of response	17 (54.8)
Non-hematologic adverse events	9 (29.0)
Cytopenia	4 (12.9)
Patient request	1 (3.2)
**Median time to discontinuation (range)**	6 months (0.8–78)
**Treatment cessation due to deep molecular remission**	2 (2.0)

*Among the 31 patients who discontinued ponatinib, the starting dose was 45 mg in 9 patients, 30 mg in 13 patients, and 15 mg in 9 patients

### Clinical outcomes

**Fig. 1:**
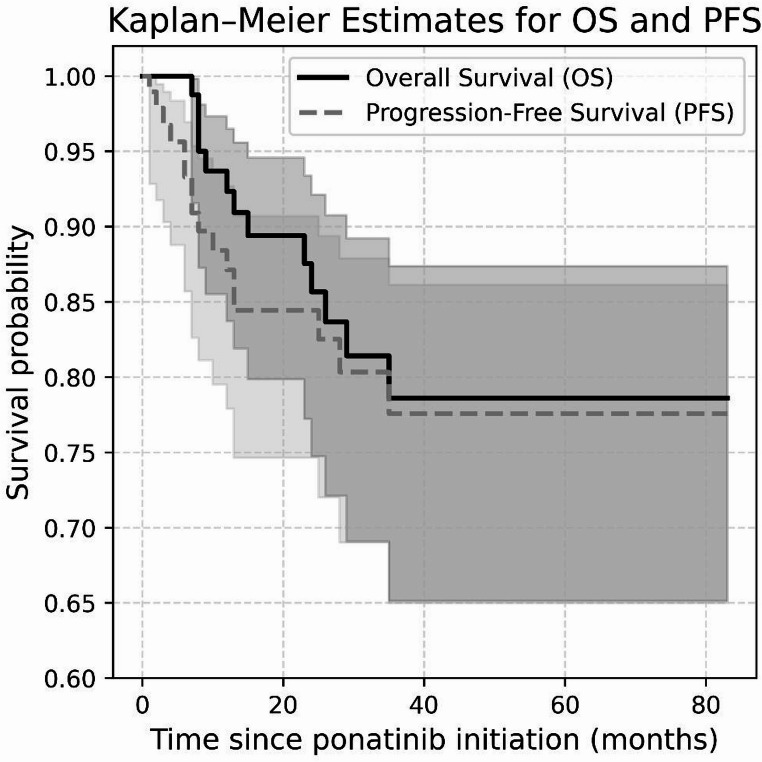
Kaplan-Meier estimates of overall survival and progression-free survival among 99 CML patients treated with ponatinib (Shaded areas represent 95% confidence intervals)

A total of eight patients (8.1%) underwent allogeneic stem cell transplantation (allo-SCT) after a median of 5 months (range: 4–15 months) of ponatinib therapy. One transplanted patient, who was in lymphoid blast phase at the time of transplantation, died 3 months post-transplant. During the study period, 14 of 99 patients (14.1%) experienced disease progression, with a median time to progression of 7 months (range, 1–28 months) following ponatinib initiation. Nine of these patients were in the blast phase at the time of progression. The estimated 2-year progression-free survival (PFS) rate was 84.4%. At the last follow-up, 13 patients (13.1%) had died, with a median time to death of 13 months (range: 7–35 months) after starting ponatinib. The estimated 2-year overall survival (OS) rate was 85.7% (Fig. 1). The most common causes of death were CML progression (*n* = 6) and infections/sepsis (*n* = 4), while single cases of multi-organ failure, age-related decline, and unknown causes were also reported.

## Discussion

The PONDEROSA study provides insights from clinical practice into the effectiveness and safety of ponatinib in patients with CML in Germany and the Czech Republic. These findings confirm ponatinib’s role as an effective treatment option in patients with resistant disease or prior multi-line TKI failure, particularly those harboring the *BCR::ABL1* T315I mutation. The present results compare well with other registry studies, where MMR rates ranged between 40-60% in patients with advanced prior treatment histories [15–19]. Notably, the French TOPASE registry reported that 73.5% of chronic-phase CML patients with at least one efficacy evaluation achieved MMR or a deeper response at least once during the study period. This rate is higher than in most other registries and may reflect differences in patient characteristics or treatment strategies [20]. In addition to these differences in response rates, PONDEROSA provides several unique features compared with previously published real-world registries. It represents the first multicenter observational dataset from Germany and the Czech Republic, reflecting routine clinical practice in Central Europe. Moreover, unlike registries, which were purely retrospective, PONDEROSA combines both retrospective and prospective data collection. This mixed design allowed the capture of treatment decisions across different time periods, including the increasing use of lower ponatinib starting doses in routine care. Careful dose adjustment of ponatinib is essential to enhance therapeutic benefit while reducing toxicity risk. The OPTIC trial demonstrated that starting at 45 mg/day and reducing to 15 mg/day upon response could maintain efficacy while reducing AOEs, with exposure-adjusted AOE rates of 9.6%, 5.3%, and 3.2% in the 45 mg, 30 mg, and 15 mg cohorts, respectively [6, 8]. A comparative analysis of the PACE and OPTIC trials showed a 60% lower risk of AOEs in OPTIC compared to PACE, based on propensity score weighting [7]. In addition, the prospective PONS trial evaluated ponatinib at a reduced starting dose of 30 mg/day as second-line treatment for chronic-phase CML patients after failure or intolerance to a first-line second-generation TKI. In a cohort selected for low cardiovascular risk and monitored with rigorous cardiological assessment, no serious cardiovascular events were observed, and the treatment led to favorable molecular responses within the first year [22]. In our study, only 32.3% of patients started at 45 mg, with 37.3% initiating at 30 mg and 29.3% at 15 mg, reflecting a shift toward lower starting doses in routine practice. 53.5% of patients required dose modifications, most commonly due to toxicity. The incidence of severe (grade 3–4) cardiovascular or cerebrovascular AEs in the present study was 12.1% (4.8% of all recorded AEs). This rate should be interpreted in the context of the underlying patient characteristics: at baseline, a relevant proportion of patients presented with cardiovascular risk factors such as arterial hypertension (42.4%) and diabetes mellitus (13.1%). Moreover, many patients had previously been treated with second-generation TKIs such as nilotinib, which are associated with long-term cardiovascular toxicity. In the TOPASE registry, 12 AOEs were reported in 11 of 120 patients (9.2%), with 8 events (6.7%) classified as severe [20]. An earlier retrospective French real-world study (PEARL) reported an overall rate of cardiovascular events of 47% (retrospective, under Ponatinib treatment), although most were mild; two cardiovascular-related deaths were observed [14]. The Belgian registry documented cardiovascular AEs in 16% of patients; however, as CTCAE grading was not systematically applied, the severity of these events remains unclear [17]. In the Argentine registry cohort, severe AOEs were observed in eight patients (10.9%) after a median treatment duration of 5 months, including coronary (60%), cerebrovascular (30%), and peripheral arterial (10%) events. Two patients in that study died due to AOEs [19], while in the PONDEROSA cohort, no cardiovascular-related deaths were reported. These findings highlight that while ponatinib is associated with an increased risk of AOEs, the impact of patient selection, cardiovascular risk management, and treatment duration may influence clinical outcomes. Importantly, in our study, adjusted dosing strategies and close monitoring may have contributed to the absence of fatal cardiovascular events. Allo-SCT remains a crucial option for patients with high-risk disease or inadequate response to TKI therapy. In the present study, 8.1% of patients underwent allo-SCT, underscoring its role in managing advanced or resistant CML. Ponatinib was effectively used to stabilize disease prior to transplantation.

In conclusion, the PONDEROSA registry confirms the efficacy of ponatinib under routine care conditions, validating its role in heavily pretreated patients, particularly those with resistance or intolerance to prior TKIs or harboring the T315I mutation. These data also support its use as a bridging therapy for allo-SCT candidates and emphasize the importance of individualized dosing to reduce cardiovascular risks.

## Supplementary Information

Below is the link to the electronic supplementary material.

Supplementary Material 1

## Data Availability

The datasets generated during and/or analyzed during the current study are available from the corresponding author on reasonable request.
