# Supplementary material for: Ponatinib for CML patients in routine clinical practice: the PONDEROSA study
Source: Ann Hematol. 2026 Jan 15;105(1):9. doi: 10.1007/s00277-026-06788-6 (PMC12804319; doi:10.1007/s00277-026-06788-6)
Supplement: Supplementary file 1 — Supplementary Material 1 [file 277_2026_6788_MOESM1_ESM.docx]

**Ponatinib for CML Patients in Routine Clinical Practice: The PONDEROSA Study.**

Annals of Hematology

**Authors:**

Thomas Schenk^1^, Christian Fabisch^1^, Thomas Ernst^1^, Philipp Ernst^1^, Susanne Saussele^2^, Daniela Žáčková^3^, Jiří Mayer^3^, Hana Klamová^4^, Andreas Hochhaus^1^,

for the German CML Study Group and the Czech Leukemia Study Group.

^1^ Abteilung Hämatologie und Internistische Onkologie, Klinik für Innere Medizin II, Universitätsklinikum Jena; Comprehensive Cancer Center Central Germany Jena/Leipzig – Campus Jena, Jena, Germany

^2^ Medizinische Klinik III, Medizinische Fakultät Mannheim der Universität Heidelberg, Mannheim, Germany

^3^ Department of Internal Medicine, Hematology and Oncology, University Hospital Brno and Masaryk University, Brno, Czech Republic

^4^ Institute of Hematology and Blood Transfusion, Praha, Czech Republic

Corresponding author:

Dr. Thomas Schenk

Abteilung Hämatologie und Internistische Onkologie

Klinik für Innere Medizin II

Universitätsklinikum Jena

Am Klinikum 1, 07747 Jena, Germany

Phone: +49 3641 9324201; Fax: +49 3641 9324202

E-Mail: [thomas.schenk@med.uni-jena.de](mailto:thomas.schenk@med.uni-jena.de)

**Supplementary Table S1. List of investigators who participated in the PONDEROSA study**

| **Last name** | **First name** | **City** | **Institution** |
| --- | --- | --- | --- |
| Al-Ali | Haifa | Halle | Universitätsklinikum Halle |
| Becker | Martin | Porta Westfalica | Onkologische Schwerpunktpraxis |
| Bock | Torsten | Wittenberge | Gemeinschaftspraxis Dres. Bock |
| Eckert | Robert | Esslingen | Onkologische Schwerpunktpraxis |
| Faber | Edgar | Olomouc | University Hospital Olomouc,  Czech Republic |
| Franke | Georg-Nikolaus | Leipzig | Universitätsklinikum Leipzig |
| Göthert | Joachim | Essen | Universitätsklinikum Essen |
| Hackanson | Björn | Augsburg | Universitätsklinikum Augsburg |
| Hamm | Thomas | Soest | Onkologiezentrum Soest |
| Hentrich | Marcus | München | Rotkreuzklinikum München |
| Hochhaus | Andreas | Jena | Universitätsklinikum Jena |
| Hubmann | Max | Wolfratshausen | Onkologische Praxis Wolfratshausen |
| Jentsch-Ullrich | Kathleen | Magdeburg | Gemeinschaftspraxis für Hämatologie und Onkologie |
| Josting | Andreas | Berlin | Internistische FA-Praxis Prof. Josting |
| Klamová | Hana | Praha | Institute of Hematology and Blood Transfusion Prague, Czech Republic |
| Kubin | Thomas | Traunstein | Klinikum Traunstein |
| La Rosée | Paul | Villingen-Schwenningen | Klinikum der Stadt Villingen-Schwenningen |
| Mayer | Jiří | Brno | University Hospital Brno, Czech Republic |
| Reichert | Dietmar | Westerstede | Gemeinschaftspraxis für Hämatologie und Onkologie |
| Saußele | Susanne | Mannheim | Universitätsmedizin Mannheim |
| Schmidt | Burkhard | München | Hämatologisch-Onkologische Praxisgemeinschaft |
| Schmidt | Christian | Greifswald | Universitätsmedizin Greifswald |
| Schöttker | Björn | Würzburg | Hämatologisch-Onkologische Schwerpunktpraxis |
| Springer | Gregor | Stuttgart | Onkologische Praxis und Tagesklinik |
| Stegelmann | Frank | Ulm | Universitätsklinikum Ulm |
| Steinmetz | Tilmann | Köln | Gemeinschaftspraxis für Hämatologie und Onkologie |
| Südhoff | Thomas | Passau | Klinikum Passau |
| Tesch | Hans | Frankfurt/M | Centrum für Hämatologie und Onkologie Bethanien |
| Voglová | Jaroslava | Hradec Králové | University Hospital Hradec Králové, Czech Republic |
| Wolff | Thomas | Hamburg | OncoResearch Lerchenfeld GmbH |
| Zeth | Matthias | Witten | MVZ des Marien-Hospital Witten |
